# Acetylcholine-Induced Inhibition of Presynaptic Calcium Signals and Transmitter Release in the Frog Neuromuscular Junction

**DOI:** 10.3389/fphys.2016.00621

**Published:** 2016-12-12

**Authors:** Eduard Khaziev, Dmitry Samigullin, Nikita Zhilyakov, Nijaz Fatikhov, Ellya Bukharaeva, Alexei Verkhratsky, Evgeny Nikolsky

**Affiliations:** ^1^Laboratory of Biophysics of Synaptic Processes, Kazan Institute of Biochemistry and Biophysics, Kazan Scientific Center of the Russian Academy of SciencesKazan, Russia; ^2^Open Laboratory of Neuropharmacology, Kazan (Volga Region) Federal UniversityKazan, Russia; ^3^Institute of Applied Electrodynamics, Photonics and Living Systems, A.N. Tupolev Kazan National Research Technical UniversityKazan, Russia; ^4^Faculty of Life Sciences, University of ManchesterManchester, UK; ^5^Department of Biophysics, Kazan State Medical UniversityKazan, Russia

**Keywords:** neuromuscular synapse, calcium transient, presynaptic acetylcholine receptors, muscarinic receptors, nicotinic receptors, N-type Ca channels, quantum secretion of acetylcholine

## Abstract

Acetylcholine (ACh), released from axonal terminals of motor neurons in neuromuscular junctions regulates the efficacy of neurotransmission through activation of presynaptic nicotinic and muscarinic autoreceptors. Receptor-mediated presynaptic regulation could reflect either direct action on exocytotic machinery or modulation of Ca^2+^ entry and resulting intra-terminal Ca^2+^ dynamics. We have measured free intra-terminal cytosolic Ca^2+^ ([Ca^2+^]_i_) using Oregon-Green 488 microfluorimetry, in parallel with voltage-clamp recordings of spontaneous (mEPC) and evoked (EPC) postsynaptic currents in post-junctional skeletal muscle fiber. Activation of presynaptic muscarinic and nicotinic receptors with exogenous acetylcholine and its non-hydrolized analog carbachol reduced amplitude of the intra-terminal [Ca^2+^]_i_ transients and decreased quantal content (calculated by dividing the area under EPC curve by the area under mEPC curve). Pharmacological analysis revealed the role of muscarinic receptors of M_2_ subtype as well as d-tubocurarine-sensitive nicotinic receptor in presynaptic modulation of [Ca^2+^]_i_ transients. Modulation of synaptic transmission efficacy by ACh receptors was completely eliminated by pharmacological inhibition of N-type Ca^2+^ channels. We conclude that ACh receptor-mediated reduction of Ca^2+^ entry into the nerve terminal through N-type Ca^2+^ channels represents one of possible mechanism of presynaptic modulation in frog neuromuscular junction.

## Introduction

Acetylcholine (ACh) when released from nerve endings upon action potential-driven depolarization, not only triggers postsynaptic response in muscle cell, but also regulates its own secretion from presynaptic terminal (Ciani and Edwards, 1963; Parnas et al., 2000; Tomas et al., 2014). This presynaptic regulation has an important for reliability of synaptic transmission. It has been shown that various subtypes of muscarinic and nicotinic acetylcholine receptors are expressed in motor nerve endings and they modulate release of ACh into the synaptic cleft (Bowman et al., 1990; Miller, 1990; Tomas et al., 2014). The direct and indirect Ca^2+^ influx resulting from the activation of neuronal nicotinic acetylcholine receptors can modulate presynaptic neurotransmitter release (Shen and Yakel, 2009). Two subtypes of muscarinic receptors are involved in the ACh release modulation: the M_1_ receptor mediates enhancement of ACh release, while the M_2_ is involved in inhibition of release (Slutsky et al., 1999). Muscarinic agonists have been shown to decrease the number of quanta released in response to stimulation of motor nerve (Arenson, 1989; Slutsky et al., 1999; Samigullin et al., 2014). Muscarinic receptors can also be activated in the absence of stimulation during spontaneous neurotransmitter release (Kupchik et al., 2008).

Although this phenomenon has received a significant research attention in the past (Nikol'skiĭ and Giniatullin, 1979; Wessler, 1989; Macleod et al., 1994; Van der Kloot et al., 1997; Nikolsky et al., 2004), fine details of ACh-dependent presynaptic regulation remain obscure. Both direct action of ACh on exocytotic machinery (Linial et al., 1997) and inhibition of presynaptic Ca^2+^ entry (Wu and Saggau, 1997; Parnas et al., 2000; Khaziev et al., 2012) have been suggested. The latter possibility, being seemingly logical, has not been analyzed in detail. The present study was designed to investigate the role of presynaptic calcium influx in autoregulation of ACh secretion by presynaptic receptors in the frog neuromuscular junction.

## Materials and methods

### Experimental preparation and ethical approval

Experiments were performed on isolated nerve-muscle preparations of *musculus cutaneous pectoris* from the frog *Rana ridibunda*. The experimental procedures were performed in accordance with the guidelines for use of laboratory animals of Kazan Federal University and Kazan Medical University, in compliance with the NIH Guide for Care and Use of Laboratory Animals. Experimental protocols met the requirements of the European Communities Council Directive 86/609/EEC and were approved by the Ethical Committee of Kazan Medical University.

### Intracellular Ca^2+^ monitoring

Cytosolic concentration of ionized Ca^2+^ ([Ca^2+^]_i_) in nerve endings was monitored using fluorescent microfluorimetry (Tsien, 1989). Each nerve-muscle preparation was loaded with Ca^2+^-sensitive dye by soaking nerve stump in 50 mM solution of fluorescent Ca^2+^-indicator Oregon Green 488 BAPTA-1 Hexapotassium Salt (Molecular Probes, Eugene, Oregon, USA); for details of loading technique see (Peng and Zucker, 1993; Wu and Betz, 1996; Tsang et al., 2000; Samigullin et al., 2015). At the end of loading protocol, all terminals in the proximal part of the nerve trunk had sufficient levels of fluorescence. It has been estimated that the intra-terminal concentration of the probe varied between 40 and 150 μM (Suzuki et al., 2000).

The fluorescent probe by definition binds Ca^2+^ and hence may affect both [Ca^2+^]_i_ dynamics and physiology of the neuromuscular junction. Our own observations (Samigullin et al., 2015) as well as observations of others (Wu and Betz, 1996) failed to detect any appreciable influence of loaded Ca^2+^ probe on the amplitude of the postsynaptic response or on the frequency of the miniature end-plate potentials. Nonetheless we additionally performed control experiments to compare spontaneous endplate currents (mEPC) and quantal release before and after Ca^2+^ probe loading. It appeared that Ca^2+^ probe affects neither; the mEPC frequency was 0.32 ± 0.03 Hz (*n* = 4, *P* < 0.05) before and 0.32 ± 0.15 Hz (*n* = 4, *P* < 0.05) after loading of the probe; similarly the quantal content was 1.06 ± 0.09 (*n* = 3, *P* < 0.05) and 0.99 ± 0.07 (*n* = 3, *P* < 0.05). We may conclude, therefore adding Ca^2+^ probe into the cytosol of the terminal does not affect physiological parameters of neurotransmission.

Neuromuscular preparations were continuously perfused with the Ringer solution of the following content (in mM): NaCl–113, KCl–2.5, NaHCO_3_–3, MgCl_2_–6, CaCl_2_–0.9; pH was adjusted to 7.4. Low extracellular Ca^2+^ (0.9 mM) and high level of external MgCl_2_ (6 mM) were used to block muscle contraction. All experiments were performed under these conditions except the serie with decreasing extracellular Ca^2+^. Experiments were performed at 20.0 ± 0.3°C.

Fluorescent signal was recorded using photometric setup on the base of Olympus BX-51 microscope with x60 water-immersion objective connected to photodiode S1087 (Hamamatsu, Japan) as described in Sinha and Saggau (1999) and Samigullin et al. (2010, 2015). The region for recording was selected by optical viewfinder (Till Photonics, Munich, Germany). Excitation light (488 nm) was generated by Polychrome V (Till Photonics, Munich, Germany). To minimize bleaching of the dye and decrease background fluorescence, the recording area of nerve terminal was restricted by an iris diaphragm. Illumination was controlled by a shutter with a typical exposure time of 400 ms and a delivery rate of 0.5 Hz. Motor nerve was electrically stimulated by rectangular voltage pulses of 0.2 ms in duration and supra-threshold amplitude at a frequency of 0.5 Hz using the “suction” electrode described earlier (Kazakov et al., 2015). The photodiode signal was digitized by the ADC Digidata 1440A (Molecular Devices, USA) with sampling rate 10.256 kHz. Fluorescence recordings, illumination and electrical stimulation were all controlled by WinWCP software (Strathclyde University, Glasgow, UK). The peak amplitude of [Ca^2+^]_i_ transients was measured and changes in fluorescence are represented as ΔF/F_0_ (the change in fluorescence intensity relative to the background fluorescence as a percentage). For each experiment, about 60 fluorescence responses were averaged.

### Electrophysiological recordings

Spontaneous and evoked endplate currents (mEPC and EPC, respectively) were recorded with a two-electrode voltage clamp technique at a holding potential of −60 mV. Intracellular microelectrodes (5–10 MΩ in resistance) were filled with 2.5 M KCl. Currents were recorded using Axoclamp 900A amplifier and digitized by Digidata 1440A (Molecular Devices, USA) under control of Clampex software v. 10.5. Quantum content of EPCs was calculated by dividing the area under EPC curve by the area under mEPC curve.

### Chemicals

All reagents were obtained from Sigma (Saint Louis, Missouri, USA). Drugs were diluted in extracellular solution to get the following final concentrations: carbachol (10 μM), acetylcholine (100 μM), neostigmine (1 μM), atropine (1 μM), d-tubocurarine (10 μM), muscarine (10 μM), nicotine (10 μM), pirenzepine (100 nM), methoctramine (10 nM), mecamylamine (640 nM–6.9 μM), metillikakonitin (10 nM), ω-conotoxin GVIA (300 nM).

### Data analysis

Experimentally measured relative amplitudes were tested for normal distribution. Statistical significance of relative amplitudes we assessed by Student's *t*-test for pairwise variant. Then data are presented as mean (%) ± SEM. Values of *P* < 0.05 were considered significant.

## Results

### Acetylcholine and carbachol reduce [Ca^2+^]_i_ transients and quantal content of EPC

**Figure 1:**
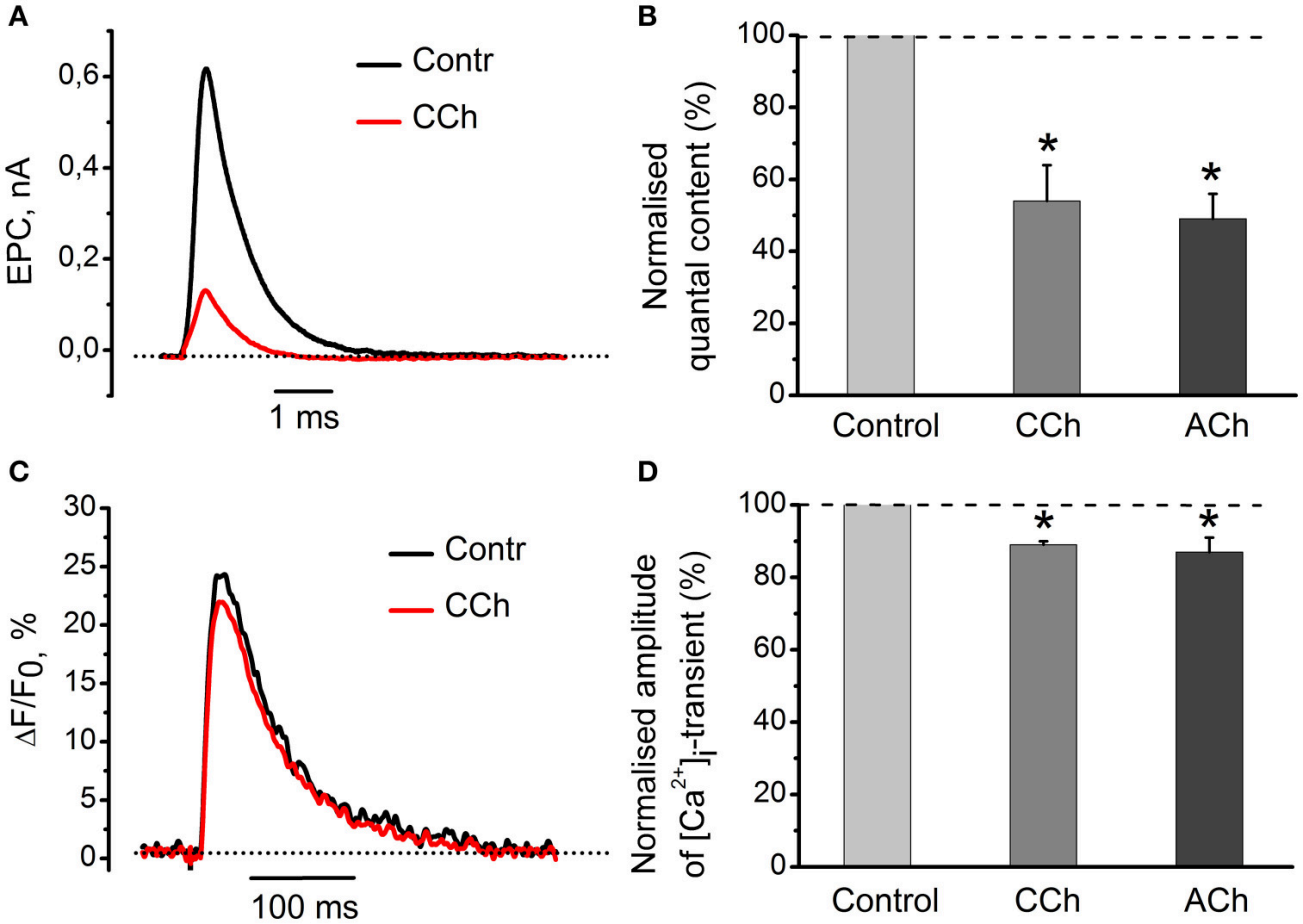
**Acetylcholine and carbachol reduce [Ca^2+^]_i_ transients and the EPC quantal content. (A)** Representative EPCs in control conditions and in the presence of 10 μM carbachol. **(B)** Quantal content in the presence of 10 μM carbachol or 100 μM ACh. **(C)** [Ca^2+^]_i_ transients in control and in the presence of 10 μM carbachol. **(D)** Amplitude of [Ca^2+^]_i_ transients in the presence of carbachol and acetylcholine as % of control (control set as100%). Abbreviations: Contr, control; CCh, carbachol; ACh, acetylcholine. ^*^P < 0.05 vs. control saline; *n* = 4–6.

Application of 10 μM of carbachol decreased amplitudes of mEPC and EPC by 26 ± 5% (*n* = 4, *P* < 0.05) and 61 ± 8% (*n* = 4, *P* < 0.05), respectively (Figure 1A). Carbachol also reduced quantal content by 46 ± 10% (*n* = 4, *P* < 0.05) as compared to control (Figure 1B). 100 μM ACh attenuated quantal content by 49 ± 7% (*n* = 18, *P* < 0.05, Figure 1B). Both carbachol and ACh also attenuated amplitude of [Ca^2+^]_i_ transients by 11 ± 1% (*n* = 5, *P* < 0.05) and 13 ± 4% (*n* = 6, *P* < 0.05,) respectively (Figures 1C,D). Since inhibitory effects of exogenous acetylcholine and carbachol were similar, we used carbachol in all subsequent experiments to activate nicotinic and muscarinic receptors simultaneously. Nicotine and muscarine, however, were used to selectively activate respective receptors types. Also, carbachol is not hydrolysed by acetylcholine esterase, and hence its concentration remains constant over the entire experiment duration.

### [Ca^2+^]_i_ transients and quantal release after changing driving force for Ca^2+^ entry into nerve terminal

**Figure 2:**
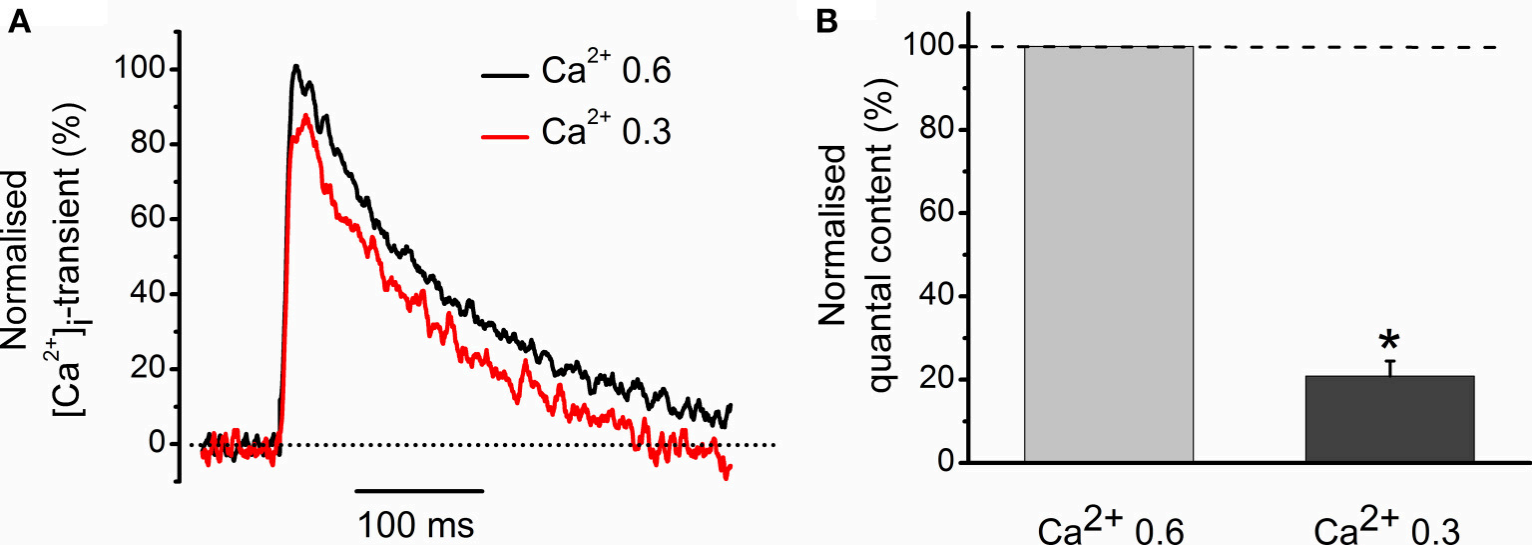
**Role of Ca^2+^ entry in generation of [Ca^2+^]_i_ transients and regulation of quantal release. (A)** [Ca^2+^]_i_ transients at two different Ca^2+^ concentrations in extracellular solution. Amplitude of [Ca^2+^]_i_ transients at 0.6 mM Ca^2+^ is set as 100%. **(B)** Average quantum content of EPC at two different concentrations of extracellular Ca^2+^. ^*^P < 0.05 vs. control saline; *n* = 3.

We were interested to elucidate whether a 11% decrease in the peak magnitude of [Ca^2+^]_i_ transient is sufficient to account for a 46% decline in neurotransmitter release observed with carbachol (Figures 1A–C). For this purpose, we have reduced the external concentration of Ca^2+^ from 0.6 to 0.3 mM and found that it decreased the evoked [Ca^2+^]_i_ transient by 23 ± 4% (*n* = 3) (Figure 2A). In parallel with this, quantal content dropped by 80 ± 4% (Figure 2B). Thus, carbachol-induced decrease in the size of evoked [Ca^2+^]_i_ transients is more than sufficient to produce inhibition of neurotransmitter release observed in our experiments earlier. Similar observations were made previously while studying quantal content and [Ca^2+^]_i_ transients under activation of cannabinoid receptors (Newman et al., 2007).

### [Ca^2+^]_i_ transient modulation by muscarine and nicotine

**Figure 3:**
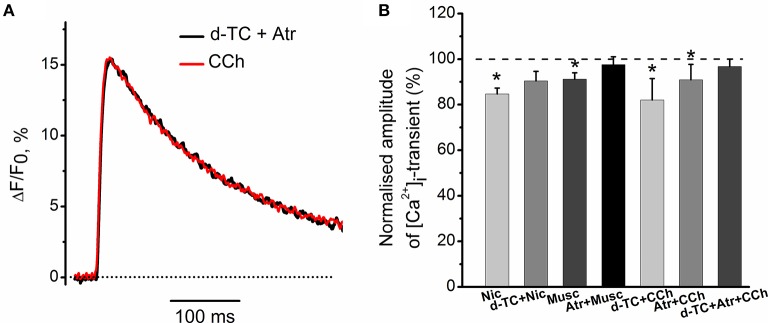
**Modulation of [Ca^2+^]_i_ transient by muscarine and nicotine. (A)** [Ca^2+^]_i_ transient in the presence of carbachol (10 μM) after pre-treatment with the mixture of atropine (1 μM) and d-tubocurarine (10 μM). **(B)** Average amplitudes of [Ca^2+^]_i_ transient normalized to the control in the presence of nicotine (10 μM); in the presence of nicotine after pre-treatment with d-tubocurarine (10 μM); in the presence of muscarine (10 μM); in the presence of muscarine after pre-treatment with atropine (1 μM), in the presence of carbachol (10 μM) after pre-treatment with d-tubocurarine (10 μM), in the presence of carbachol (10 μM) after pre-treatment with atropine (1 μM) and in the presence of carbachol after pre-treatment with the mixture of atropine (1 μM) and d-tubocurarine (10 μM). Abbreviations: d-TC, d-tubocurarine; Atr, atropine; CCh, carbachol; Nic, nicotine (10 μM); Musc, muscarine (10 μM). ^*^P < 0.05 vs. control saline; *n* = 4–7.

Exposure to 10 μM nicotine reduced the amplitude of [Ca^2+^]_i_ transient by 15 ± 3% (*n* = 6, *P* < 0.05, Figure 3B). Application of 10 μM muscarine decreased the amplitude of [Ca^2+^]_i_ transient by 8 ± 2% (*n* = 5, *P* < 0.05, Figure 3B). Nicotinic antagonist d-tubocurarine (10 μM) partially but significantly reduced nicotine effect on the [Ca^2+^]_i_ transient, while incubation with muscarinic blocker atropine (1 μM) completely eliminated presynaptic effect of muscarine (Figure 3B). In the presence of atropine (1 μM), carbachol (10 μM) reduced [Ca^2+^]_i_ transient by 9 ± 5% (*n* = 6, *P* < 0.05, Figure 3B), while in the presence of d-tubocurarine (10 μM)—by 18 ± 9% (*n* = 4, *P* < 0.05, Figure 3B). Pre-incubation with the mixture of d-tubocurarine (10 μM) and atropine (1 μM), completely eliminated carbachol effects on [Ca^2+^]_i_ transients (Figures 3A,B).

**Figure 4:**
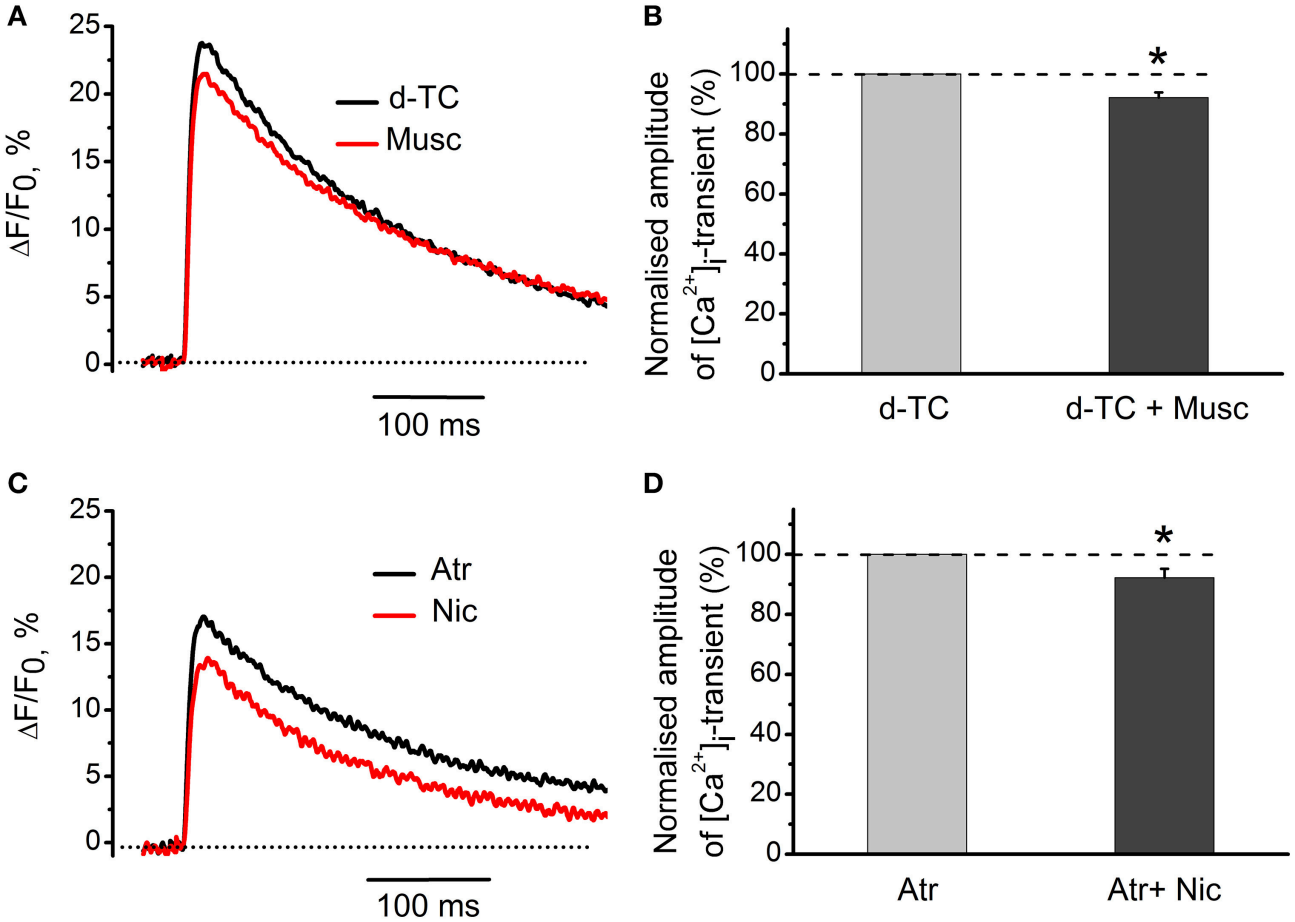
**Specific effects of activation of muscarinic and nicotinic receptors on [Ca^2+^]_i_ transients. (A)** [Ca^2+^]_i_ transients in the presence of muscarine (10 μM) after blockade of nicotinic receptors by d-tubocurarine (10 μM). **(B)** Average amplitude of [Ca^2+^]_i_ transient in the presence of muscarine (10 μM) after blockade of nicotinic receptors by tubocurarine (10 μM). **(C)** Amplitude of [Ca^2+^]_i_ transient in the presence of nicotine (10 μM) after blockade of muscarinic receptors by atropine (1 μM). **(D)** Average amplitude of [Ca^2+^]_i_ transient in presence of nicotine when muscarinic receptors are blocked by atropine. Abbreviations: d-TC, d-tubocurarine; Atr, atropine; Nic, nicotine; Musc, muscarine. ^*^P < 0.05; *n* = 4–6.

Nicotinic blocker d-tubocurarine did not change the inhibitory effect of muscarine alone—it reduced [Ca^2+^]_i_ transients by 8 ± 2% (*n* = 7, *P* < 0.05, Figures 4A,B). Similarly, blockade of muscarinic receptors by atropine did not significantly affect the inhibitory action of nicotine –[Ca^2+^]_i_ transients were reduced by 8 ± 3% (*n* = 7, *P* < 0.05, Figures 4C,D).

### Identification of muscarinic receptor subtypes that mediate the effects of cholinomimetics

**Figure 5:**
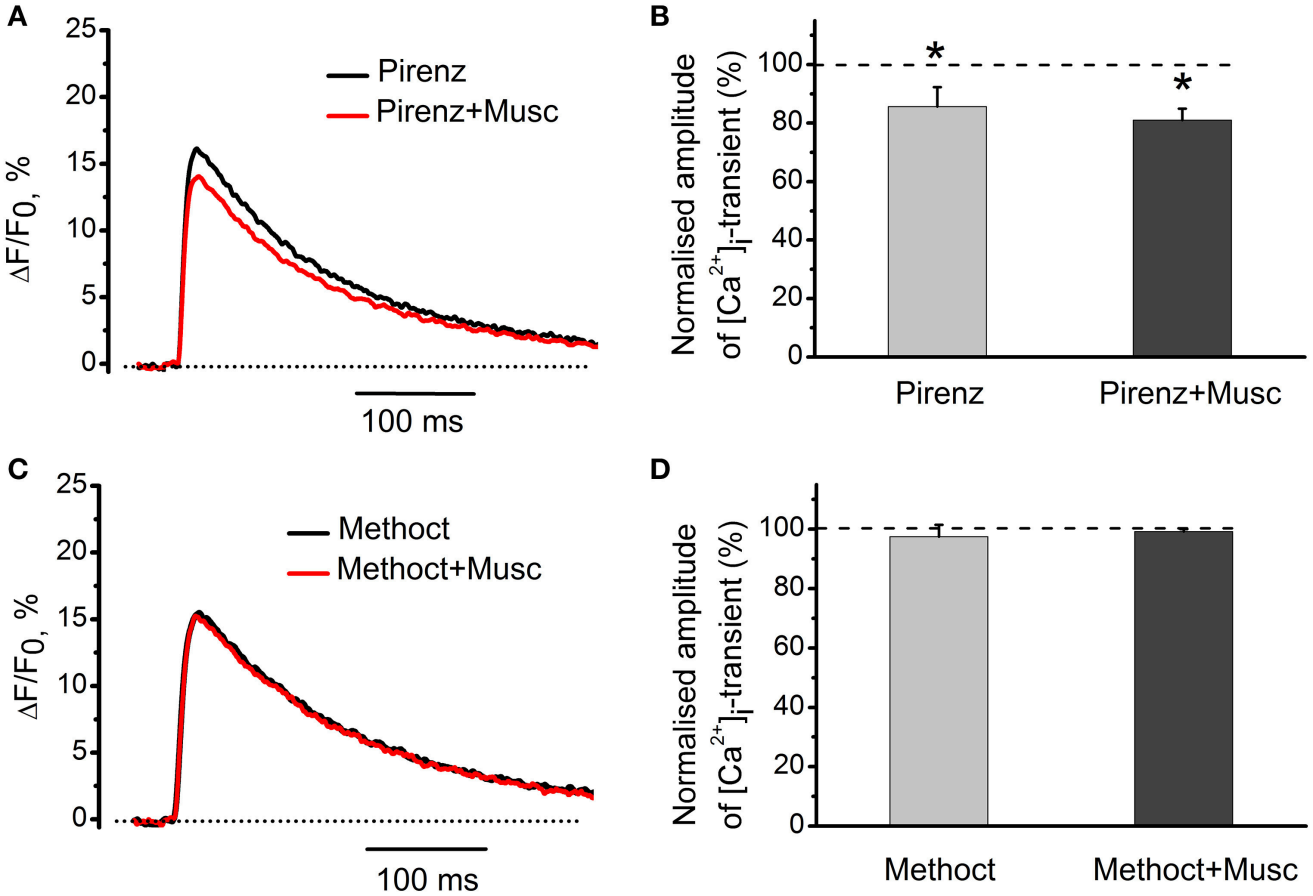
**Identification of muscarinic receptor subtypes mediating effects of cholinomimetics. (A)** [Ca^2+^]_i_ transients in the presence of muscarine (10 μM) and pirenzepine (100 nM). **(B)** Mean values of amplitude of [Ca^2+^]_i_ transients in the presence of muscarine and pirenzepine. **(C)** [Ca^2+^]_i_ transients in the presence of methoctramine (10 nM) and muscarine (10 μM). **(D)** Mean values of amplitude of [Ca^2+^]_i_ transients in the presence of methoctramine (10 nM) and muscarine (10 μM). Abbreviations: Pirenz, pirenzepine; Musc, muscarine; Methoct, methoctramine. ^*^P < 0.05 vs. control; *n* = 5.

M_1_-receptor blocker pirenzepine (100 nM) by itself decreased [Ca^2+^]_i_ transients by 14 ± 7% (*n* = 5, *P* < 0.05, Figures 5A,B). Addition of muscarine further decreased the amplitude of [Ca^2+^]_i_ transient (Figures 5A,B). Exposure to 10 nM methoctramine (specific blocker of muscarinic M_2_ receptor subtypes), did not affect [Ca^2+^]_i_ transients by itself. In the presence of methoctramine, however, the inhibitory action of muscarine on [Ca^2+^]_i_ transient was completely eliminated (Figures 5C,D).

### Identification of nicotinic receptor subtypes that mediate the effects of cholinomimetics

**Figure 6:**
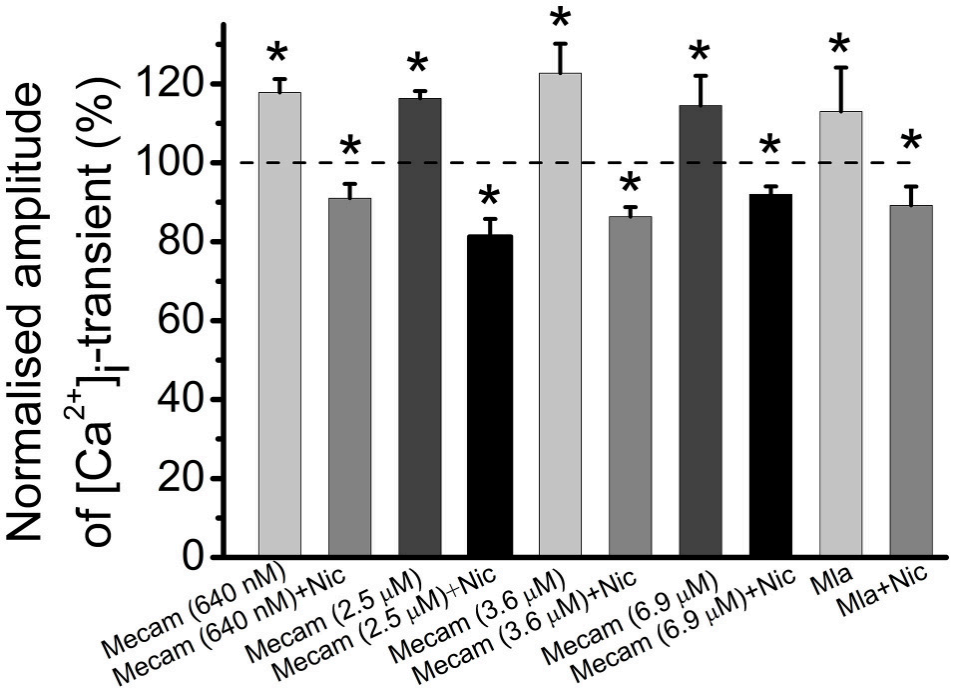
**Identification of nicotinic receptor subtypes mediating effects of cholinomimetics**. Mean values of magnitude of [Ca^2+^]_i_ transients in the presence of different concentrations (they are indicated in brackets) of mecamylamine blocking various subtypes of nicotinic receptors and after addition of nicotine (10 μM); in the presence of metillikakonitin (10 nM) and after addition of nicotine (10 μM). Abbreviations: Nic, nicotine; Mecam, mecamylamine. MLA, metillikakonitin. Control corresponds to 100%. ^*^P < 0.05 vs. control saline; *n* = 3–6.

In these experiments nicotine was used as a specific agonist of nicotinic acetylcholine receptors. Mecamylamine is able to block various subtypes of nicotinic receptors depending on concentration within the range from 640 nM to 6.9 μM (Papke et al., 2001; Rabenstein et al., 2006; Ostroumov et al., 2008). Next, we tested the effects of different concentrations of mecamylamine. Application of 640 nM mecamylamine increased [Ca^2+^]_i_ transient amplitude by 18 ± 3% (*n* = 5, *P* < 0.05, Figure 6). When 640 nM mecamylamine was applied with nicotine [Ca^2+^]_i_ transient amplitude decreased by 9 ± 4% (*n* = 5, *P* < 0.05, Figure 6). At 2.5 μM, mecamylamine increased [Ca^2+^]_i_ transient amplitude by 16 ± 2% (*n* = 3, *P* < 0.05, Figure 6), while subsequent addition of nicotine reduced [Ca^2+^]_i_ transient amplitude by 19 ± 4% (*n* = 3, *P* < 0.05, Figure 6). At 3.6 μM mecamylamine increased [Ca^2+^]_i_ transient by 23 ± 7% (*n* = 6, *P* < 0.05, Figure 6), whereas its combined application with nicotine decreased [Ca^2+^]_i_ transient by 14 ± 2% (*n* = 6, *P* < 0.05, Figure 6). Finally, at 6.9 μM, mecamylamine increased [Ca^2+^]_i_ transient by 15 ± 8% (*n* = 3, *P* < 0.05, Figure 6), while combined application with nicotine decreased [Ca^2+^]_i_ transient amplitude by 8 ± 2% (*n* = 3, *P* < 0.05, Figure 6). Metillikakonitin is a specific blocker of the α7-nicotinic receptor subunit. Adding metillikakonitin at 10 nM increased the amplitude of [Ca^2+^]_i_ transient by 13 ± 11% (*n* = 5, *P* < 0.05, Figure 6), while subsequent addition of nicotine decreased [Ca^2+^]_i_ transients by 11 ± 5% (*n* = 6, *P* < 0.05, Figure 6).

### Voltage-gated Ca^2+^ channels involved in carbachol modulation of [Ca^2+^]_i_ dynamics

**Figure 7:**
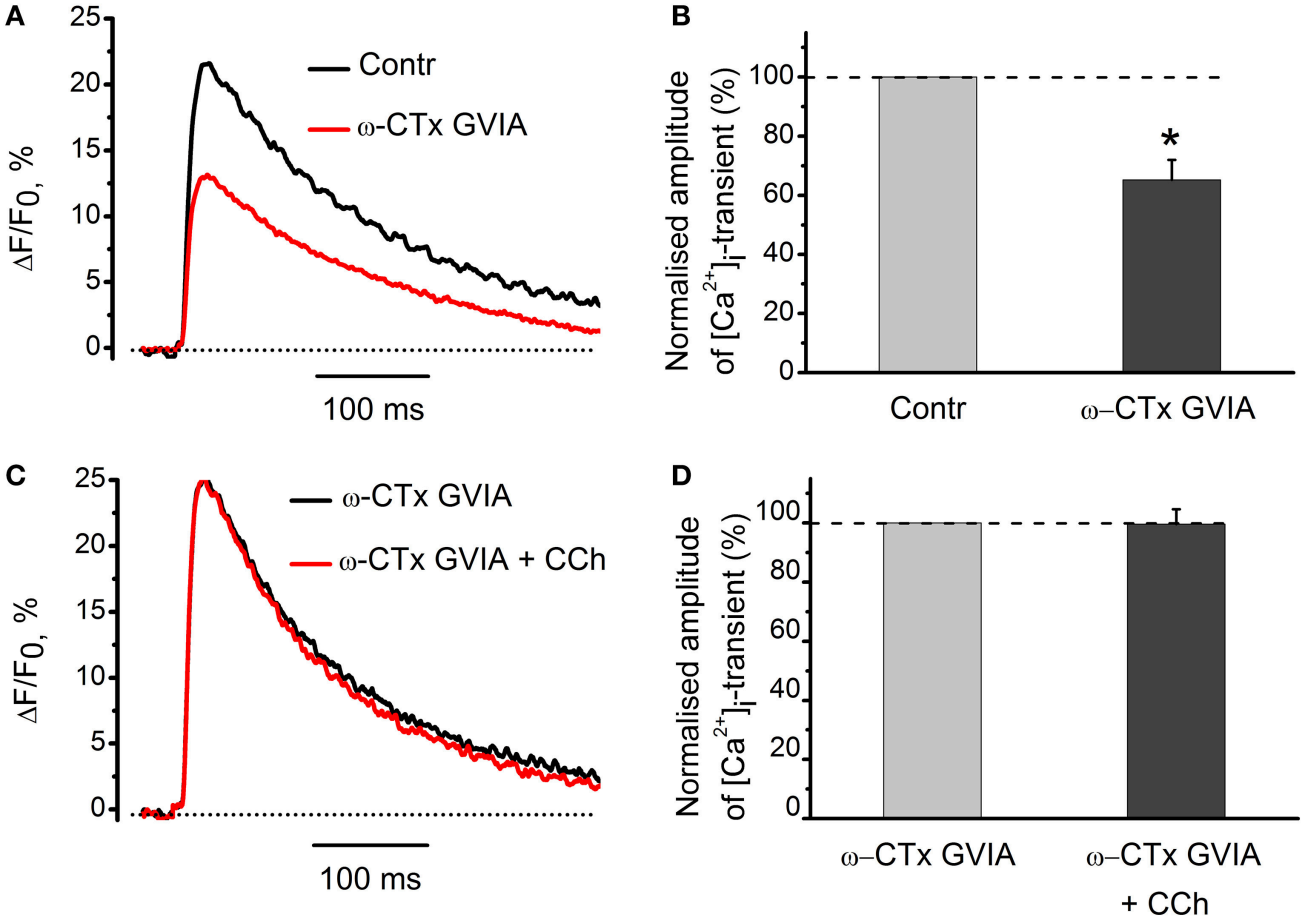
**The role of voltage-gated Ca^2+^-channels in the effects of carbachol on [Ca^2+^]_i_ dynamics. (A)** [Ca^2+^]_i_ transients in the presence of specific blocker of N-type Ca^2+^ channels, ω-conotoxin GVIA (300 nM). **(B)** Mean values of amplitudes of [Ca^2+^]_i_ transients normalized to control. **(C)** Effect of carbachol (10 μM) on [Ca^2+^]_i_ transients in the presence of ω-conotoxin GVIA. **(D)** Mean values of amplitudes of [Ca^2+^]_i_ transients. Abbreviations: Contr, control; ω-CTx GVIA, ω-conotoxin GVIA; CCh, carbachol. ^*^P < 0.05; *n* = 5.

Since both acetylcholine and carbachol reduce [Ca^2+^]_i_ transient and quantum content of endplate currents, we suggested that cholinergic modulation of neurotransmitter release could result from attenuated Ca^2+^ entry into motor nerve endings (Khaziev et al., 2012). In this work, we found that application of specific N-type Ca^2+^ channel blocker, ω-conotoxin GVIA (300 nM) reduced [Ca^2+^]_i_ transient by 35 ± 7% (*n* = 5, *P* < 0.05, Figures 7A,B). In the presence of ω-conotoxin GVIA carbachol became ineffective (Figures 7C,D), indicating that N-type Ca^2+^-channels play role in presynaptic modulation observed.

### Action of antagonists of nicotinic and muscarinic acetylcholine receptors on [Ca^2+^]_i_ dynamics in the absence of exogenous cholinomimetics

**Figure 8:**
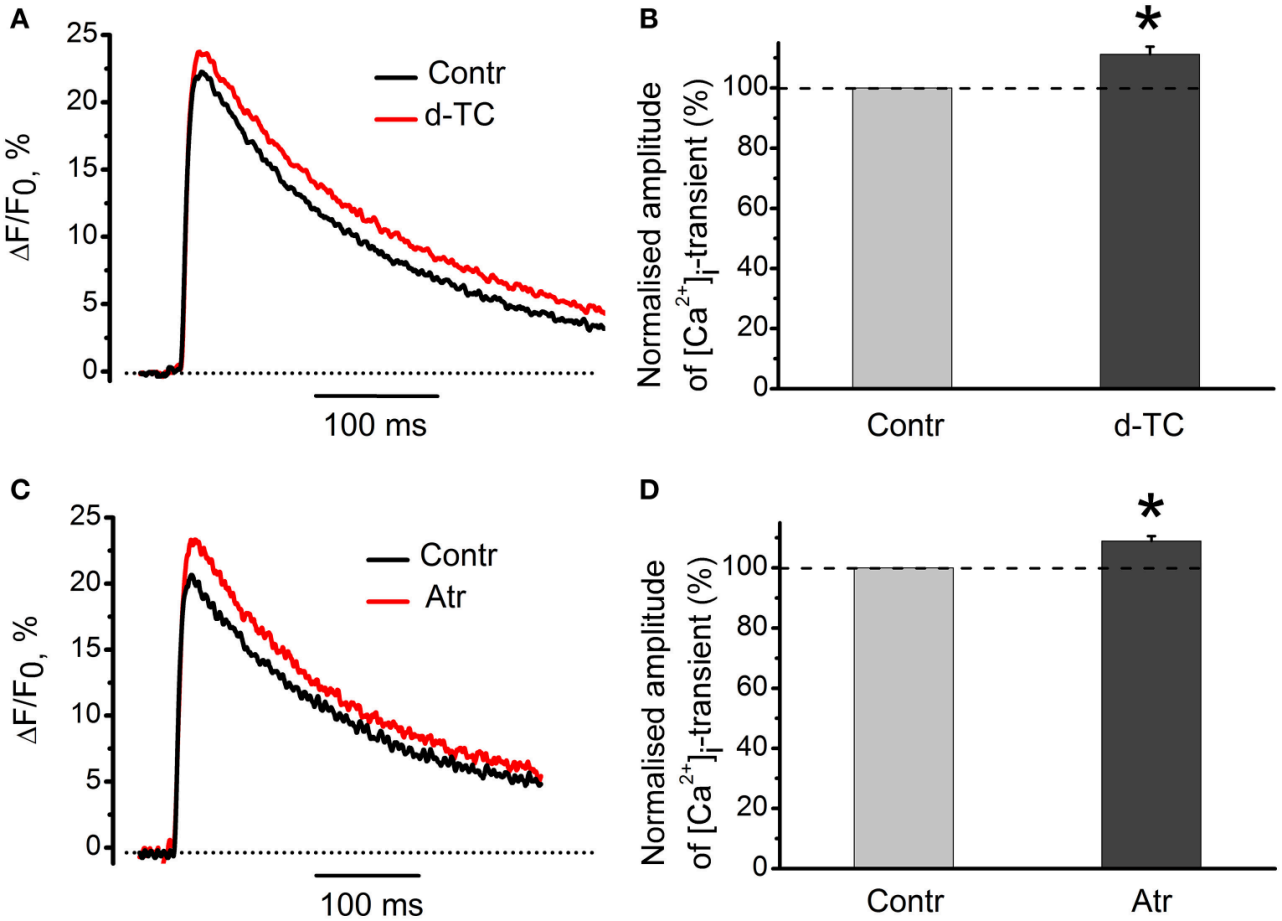
**Action of antagonists of nicotinic and muscarinic acetylcholine receptors on [Ca^2+^]_i_-dynamics in the absence of exogenous cholinomimetics. (A)** [Ca^2+^]_i_ transients in control and in the presence of d-tubocurarine (10 μM); **(B)** mean values of amplitudes of [Ca^2+^]_i_ transients in the presence of d-tubocurarine; **(C)** [Ca^2+^]_i_ transients in control and in the presence of atropine (1 μM); **(D)** mean values of amplitudes of [Ca^2+^]_i_ transients in the presence of atropine. Abbreviations: Contr, control; d-TC, d-tubocurarine; Atr, atropine. ^*^P < 0.05 vs. control; *n* = 7–15.

Non-specific blocker of all types of nicotinic receptors, d-tubocurarine (10 μM) increased the amplitude of [Ca^2+^]_i_ transients by 11 ± 3% (*n* = 15, *P* < 0.05, Figures 8A,B). Non-specific blocker of muscarinic receptors, atropine (1 μM) also increased [Ca^2+^]_i_ transient by 9 ± 2% (*n* = 19, *P* < 0.05, Figures 8C,D). When applied together, d-tubocurarine and atropine increased the amplitude of [Ca^2+^]_i_ transients by 7 ± 2% (*n* = 7, *P* < 0.05), indicating the absence of additive facilitating effects on [Ca^2+^]_i_ transient. An increase in Ca^2+^-response in the presence of nicotinic or muscarinic acetylcholine receptor blockers suggests the presence of some tonic concentration of endogenous acetylcholine in the synaptic cleft. This acetylcholine interacts with nicotinic and muscarinic receptors and causes inhibition of Ca^2+^-ions entry into nerve terminal.

### Effects of acetylcholinesterase inhibitor neostigmine on [Ca^2+^]_i_ dynamics

**Figure 9:**
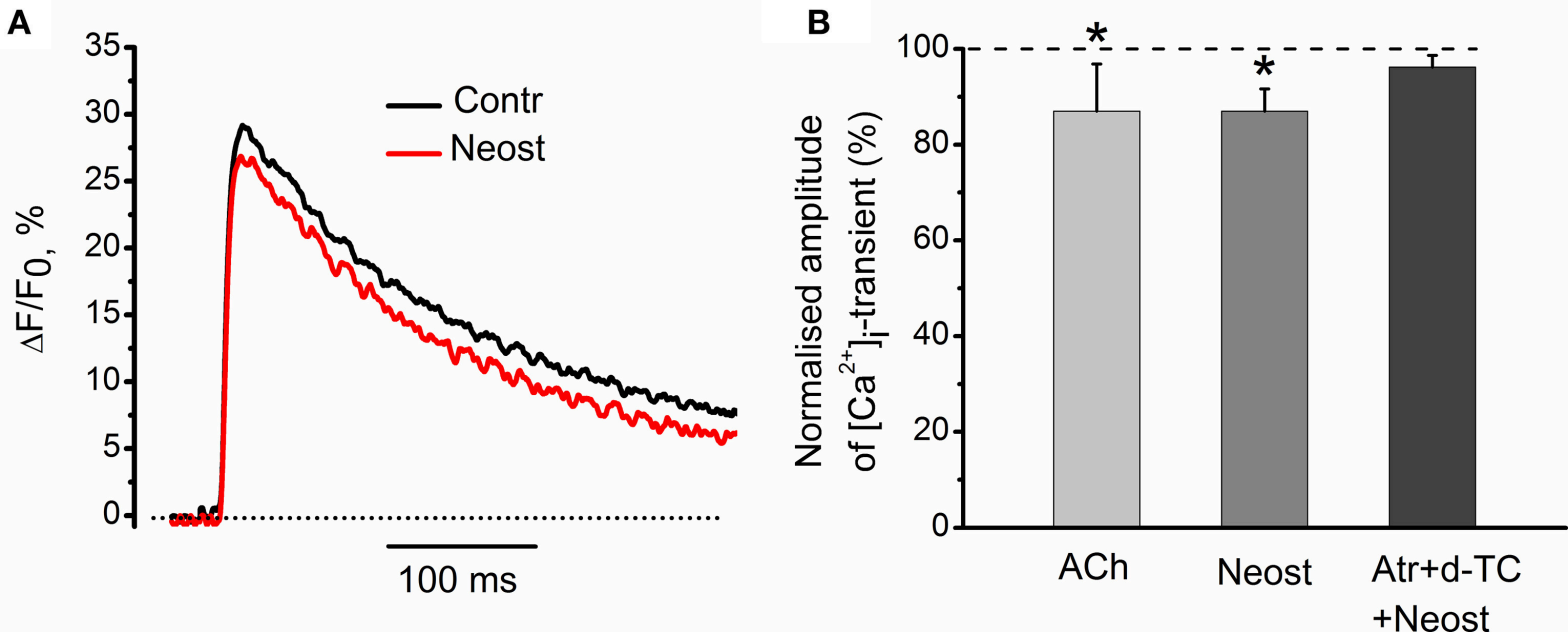
**Effects of acetylcholinesterase inhibitor neostigmine on [Ca^2+^]_i_-dynamics. (A)** [Ca^2+^]_i_ transients in control and in the presence of neostigmine (1 μM). **(B)** Mean values of amplitude of [Ca^2+^]_i_ transient in the presence of acetylcholine (100 μM); in the presence of neostigmine alone and after pre-treatment by d-tubocurarine (10 μM) and atropine (1 μM). Mean values of [Ca^2+^]_i_ transient amplitude normalized to control. Effect of exogenous acetylcholine is presented for comparison reasons. Mixture of d-tubocurarine and atropine, completely eliminated neostigmine effects on [Ca^2+^]_i_ transients. Abbreviations: Contr, control; Neost, neostigmine; ACh, acetylcholine; Atr, atropine (1 μM), d-TC, d-tubocurarine (10 μM). ^*^P < 0.05 vs. control saline; *n* = 5.

It has been shown earlier that anticholinesterase drugs lead to accumulation of endogenous acetylcholine in synaptic cleft (Loewi and Hellauer, 1938; Fatt and Katz, 1952; Fedorov, 1976). At low frequencies of motor nerve stimulation, acetylcholinesterase inhibitor, neostigmine (1 μM), decreased [Ca^2+^]_i_ transients by 13 ± 5% (*n* = 5, *P* < 0.05, Figures 9A,B). This inhibitory effect of neostigmine was fully reversed in the presence of blockers of nicotinic and muscarinic receptors (Figure 9B). We can therefore suggest that endogenous acetylcholine modulates intra-terminal [Ca^2+^]_i_ transients by acting on presynaptic nicotinic and muscarinic acetylcholine receptors.

## Discussion

Neurotransmitter release from frog nerve endings is modulated by cholinomimetics: by acting through nicotinic receptors, they change kinetics of secretion and its quantum content, while by activation of muscarinic receptors they contribute only to regulation of quantum content (Ciani and Edwards, 1963; Nikolsky et al., 2004). Both quantum content and kinetics of neurotransmitter secretion directly depend on the level of [Ca^2+^]_i_ in presynaptic terminal (Katz and Miledi, 1965; Nikol'skiĭ et al., 2000; Samigullin et al., 2005). Hence, modulation of Ca^2+^ entry could be a plausible mechanism accounting for inhibitory effects of cholinomimetics on quantum secretion. To test this hypothesis, we monitored presynaptic [Ca^2+^]_i_ transients while pharmacologically manipulating acetylcholine receptors and Ca^2+^ channels. In our experiments the Ca^2+^ probe was loaded through the nerve stump, as described by Peng and Zucker (1993) and Wu and Betz (1996). At the end of loading protocol, all terminals in the proximal part of the nerve trunk had sufficient levels of fluorescence to allow [Ca^2+^]_i_ recordings. We (in this paper), as well as others have shown that the loading of the fluorescent dye in the nerve ending does not significantly alter the physiological parameters of secretion, such as quantal content and frequency of mEPC (Wu and Betz, 1996; Samigullin et al., 2015).

We found that carbachol and acetylcholine decreased both Ca^2+^ response and quantum content of EPCs, indicating that cholinergic modulation of neurotransmitter release can be related to changes in Ca^2+^ entry (Khaziev et al., 2012). We further showed that a relatively small decrease in the driving force for Ca^2+^ influx into nerve terminal causes significant inhibition of neurotransmitter release. This was achieved by lowering extracellular Ca^2+^ concentration from 0.6 to 0.3 mM (Figures 1, 2). This observation is in a good agreement with strong non-linear dependence of quantum content from Ca^2+^ entry into the presynaptic terminal (Dodge and Rahamimoff, 1967).

Inhibition of synaptic acetylcholinesterase by neostigmine decreased [Ca^2+^]_i_ transients, indicating that endogenous acetylcholine is able to modulate Ca^2+^ entry into the nerve ending. Exposure to d-tubocurarine and specific antagonists of nicotinic receptors (in the absence of exogenous cholinomimetics) also augmented [Ca^2+^]_i_ transient, thus indicating that tonically released ACh can block Ca^2+^ entry into the presynaptic terminal. Increase of the amplitude of [Ca^2+^]_i_ transients by atropine is in a qualitative agreement with atropine-induced increase in the secretion at frog synapses due to elimination of tonic action of endogenous acetylcholine as described earlier (Slutsky et al., 1999). We suggest that release of endogenous ACh during stimulation of the motor nerve and spontaneous quantal and non-quantal release under physiological conditions results in modulation of presynaptic Ca^2+^ entry and provides a physiologically important negative feedback (Katz and Miledi, 1965; Vyskocil et al., 2009). Presynaptic acetylcholine receptors of both muscarinic and nicotinic nature are involved in this regulation. Since blockade of M_1_-muscarinic receptor by pirenzepine reduced of [Ca^2+^]_i_ transient, we may presume that activation of these receptors could enhance Ca^2+^ entry. These results are in good agreement with previous observations indicating that activation of M_1_ receptors enhances, whereas activation of M_2_ receptors inhibits ACh release (Slutsky et al., 1999). According to our results M_2_ subtype is involved in modulation of presynaptic Ca^2+^ entry, because specific M_2_ blocker methoctramine eliminates inhibitory effect of muscarine, whereas M_1_ antagonist pirenzepine has no such effect.

Mecamylamine and metillikakonitin did not prevent the inhibitory effect of nicotine while d-tubocurarine did. This indicates that some d-tubocurarine-sensitive nicotinic receptors are involved in the presynaptic inhibitory action of endogenous acetylcholine, and inhibition of this receptor in the absence of exogenous cholinomimetics enhances [Ca^2+^]_i_ transients. Specific N-type Ca^2+^-channel antagonist conotoxin GVIA removes inhibition of [Ca^2+^]_i_ transients by carbachol, pointing to a primary role of N-type channels in providing Ca^2+^ entry into the nerve terminal. Furthermore, our data about the role for N-type of Ca^2+^ channels are fully compatible with earlier results of Van der Kloot et al. (1997) who demonstrated that ω-conotoxin eliminated effects of cholinomimetics on quantal content in frog synapses. We conclude that in the frog neuromuscular synapse activation of presynaptic M_2_ muscarinic and d-tubocurarine-sensitive nicotinic acetylcholine receptors by exogenous cholinomimetics and/or endogenous acetylcholine decreases quantal content of mediator secretion by reducing the entry of Ca^2+^ ions into the nerve endings due to inhibition of voltage-gated N-type Ca^2+^ channels.

## Author contributions

EK, DS, NZ, NF, EB, and EN contributed to the study design and acquirement of ethical approval. EK, DS, NZ, NF, and EB contributed to data collection. DS, EN, and AV analyzed the data, interpreted the data, and drafted the initial manuscript. The remaining authors critically revised the manuscript. All authors approved the final version of the manuscript. DS, EB, and EN are guarantors of the manuscript and take full responsibility for the work as a whole, including the study design, access to data, and the decision to submit and publish the manuscript.

## Funding

This research has been performed with the support of the Russian Government's Program for Competitive Growth of Kazan Federal University, grant of Program of Presidium RAS 19P and the grants from the Russian Foundation for Basic Research (16-04-01051; 16-34-00817; 15-04-02983).

### Conflict of interest statement

The authors declare that the research was conducted in the absence of any commercial or financial relationships that could be construed as a potential conflict of interest.

## Abbreviations

Ach: Acetylcholine
EPC: evoked postsynaptic currents
mEPC: spontaneous postsynaptic currents.
